# Vick's Floral Guide

**Published:** 1888-02

**Authors:** 


VICK'S FLORAL GUIDE.—A silver lining to every cloud! With the short dull days of early winter come the cheery holidays and Vick's beautiful annual, and lo! spring already appears not far distant. We can almost see the greening grass and the blooming flowers. In the way of catalogue, Vick's Floral Guide is unequaled in artistic appearance, and the edition of each year that appears simply perfect, is surpassed the next. New and beautiful engravings, and three colored plates of flowers, vegetables, and grain, are features for the issue for 1888. Its lavender tinted cover, with original designs of most pleasing effects, will ensure it a prominent place in the household and library. It is in itself a treatise on horticulture, and is adapted to the wants of all who are interested in the garden or house plants. It describes the rarest flowers and the choicest vegetables. If you want to know anything about the garden, see Vick's Floral Guide, price only ten cents, including a Certificate good for ten cents worth of seeds. Published by James Vick, Seedsman, Rochester, N. Y.

